# Analysis of low-dose estrogen on callus BMD as measured by pQCT in postmenopausal women

**DOI:** 10.1186/s12891-020-03713-4

**Published:** 2020-10-19

**Authors:** K. Jäckle, J. P. Kolb, A. F. Schilling, C. Schlickewei, M. Amling, J. M. Rueger, W. Lehmann

**Affiliations:** 1 Department of Trauma Surgery, Orthopaedics and Plastic Surgery, University Medical Center Göttingen, Robert-Koch Str. 40, 37075 Göttingen, Germany; 2 Department of Trauma and Orthopaedic Surgery, University Medical Center Hamburg-Eppendorf, Martinistrasse 52, 20246 Hamburg, Germany; 3 Department of Trauma, Hand and Reconstructive Surgery, University Medical Center Hamburg-Eppendorf, Martinistrasse 52, 20246 Hamburg, Germany; 4 Center for Biomechanics and Skeletal Biology, University Medical Center Hamburg-Eppendorf, Martinistrasse 52, 20246 Hamburg, Germany

**Keywords:** Estrogen, Callus BMD as measured by pQCT, Osteoporosis, Callus formation

## Abstract

**Background:**

Osteoporosis affects elderly patients of both sexes. It is characterized by an increased fracture risk due to defective remodeling of the bone microarchitecture. It affects in particular postmenopausal women due to their decreased levels of estrogen. Preclinical studies with animals demonstrated that loss of estrogen had a negative effect on bone healing and that increasing the estrogen level led to a better bone healing. We asked whether increasing the estrogen level in menopausal patients has a beneficial effect on bone mineral density (BMD) during callus formation after a bone fracture.

**Methods:**

To investigate whether estrogen has a beneficial effect on callus BMD of postmenopausal patients, we performed a prospective double-blinded randomized study with 76 patients suffering from distal radius fractures. A total of 31 patients (71.13 years ±11.99) were treated with estrogen and 45 patients (75.62 years ±10.47) served as untreated controls. Calculated bone density as well as cortical bone density were determined by peripheral quantitative computed tomography (pQCT) prior to and 6 weeks after the surgery. Comparative measurements were performed at the fractured site and at the corresponding position of the non-fractured arm.

**Results:**

We found that unlike with preclinical models, bone fracture healing of human patients was not improved in response to estrogen treatment. Furthermore, we observed no dependence between age-dependent bone tissue loss and constant callus formation in the patients*.*

**Conclusions:**

Transdermally applied estrogen to postmenopausal women, which results in estrogen levels similar to the systemic level of premenopausal women, has no significant beneficial effect on callus BMD as measured by pQCT, as recently shown in preclinical animal models.

**Trial registration:**

Low dose estrogen has no significant effect on bone fracture healing measured by pQCT in postmenopausal women, DRKS00019858. Registered 25th November 2019 - Retrospectively registered. Trial registration number DRKS00019858.

**Supplementary information:**

The online version contains supplementary material available at 10.1186/s12891-020-03713-4.

## Background

Distal radius fractures are the most frequent bone fractures. According to reports of the World Health Organization, the total treatment cost of these fractures is among the ten most expensive medical incidents worldwide [1–3]. Elderly people, in particular postmenopausal women, are more sensitive to distal radius fractures due to osteoporosis. Osteoporosis is caused by ovarian hormone deficiency in postmenopausal women [4] and often diagnosed after ovariectomy of premenopausal women [5], suggesting that reduced ovarian hormone levels participate in the cause of the disease. In fact, morphological and metabolic studies have shown that one of the ovarian hormones, estrogen, has both anabolic and anticatabolic effects on osteoblastic and osteoclastic processes of bone remodeling [6, 7]. In preclinical model organisms, such as mice, rats and rabbits, estrogen was shown to markedly affect all phases of the bone healing process after fracture [4, 8, 9]. In mice for example, estrogen deficiency causes an impaired fracture repair with decelerated remodeling of the periosteal callus and formation of a porous neocortex [4]. Conversely, estrogen treatment of mice with bone fractures led to enhanced healing, i.e. the chondrocyte areas were larger, callus mineralization was increased, and the neocortex was thicker when compared to untreated control animals [4, 8–13]. Furthermore, estrogen treatment also led to beneficial effects on the biomechanical properties of the bones [4]. These observations are in line with the observation that osteoporotic patients sustain a prolonged and impaired healing process after bone fracture as compared to non-osteoporotic patients [14].

Encouraged by these preclinical results and the knowledge of reduced sensitivity of estrogen in old age, we decided to critically evaluate the influence of estrogen on callus BMD in a prospective randomized double-blinded and placebo-controlled trial in postmenopausal woman who suffered from a distal radius fracture. To avoid possible side effects of the hormone replacement therapy (HRT) treatment, we chose a low-dose regimen. After surgery, estrogen was applied transdermally and its effect on callus BMD was examined by peripheral quantitative computed tomography (pQCT). Using pQCT, the bone healing process can be demonstrated early and more reliable than with other methods [15].

## Methods

### Patient collective for study

**Fig. 1:**
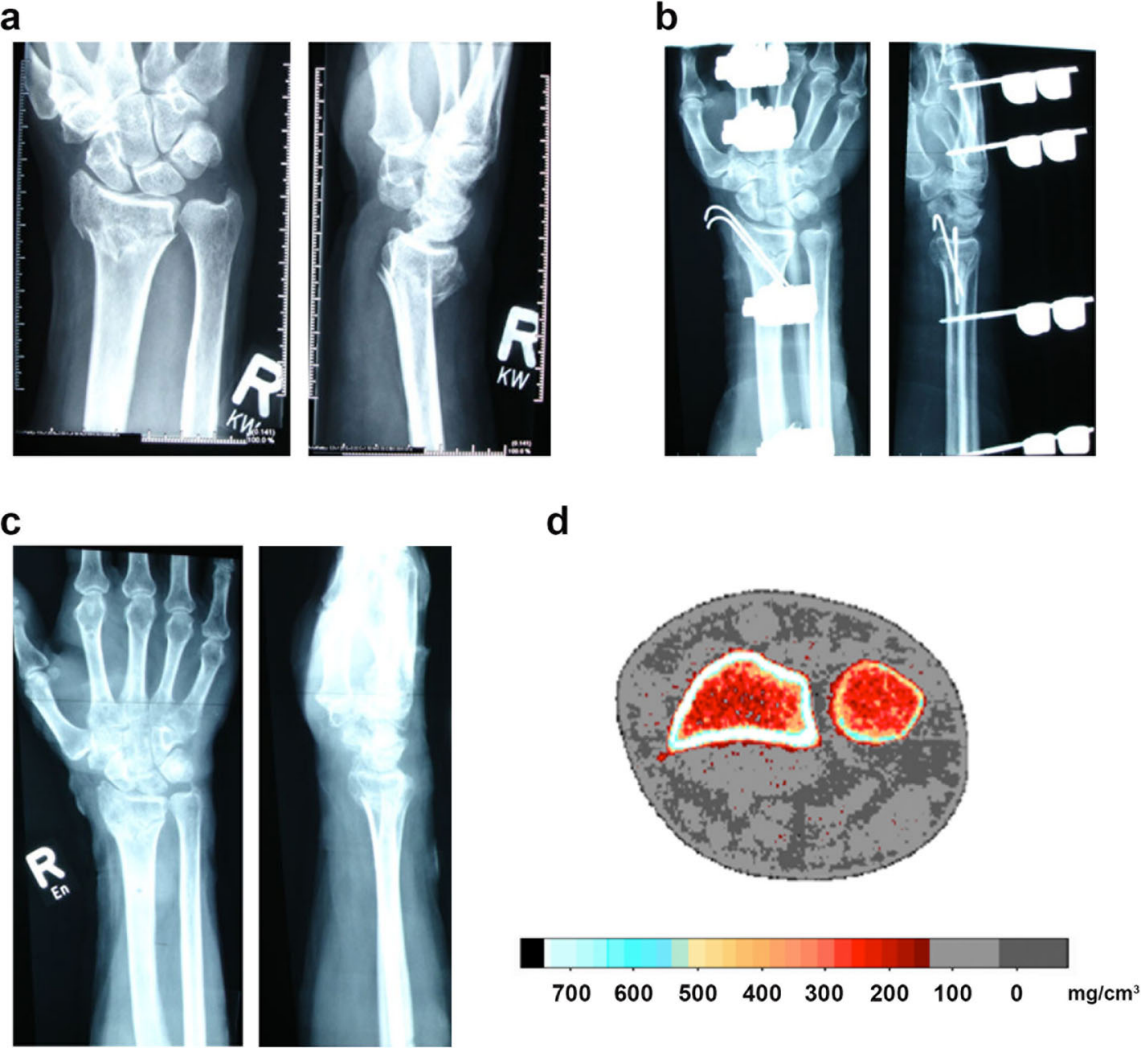
**a:** Preoperative X-ray photograph in anterior-posterior and lateral view of the right arm of the patient. **b:** Stabilisation with external fixateur and additional k-wires after 2 weeks postoperatively. **c:** Postoperative X-ray photograph in anterior-posterior and lateral view of the right arm of the patient after 6 weeks. **d:** Example of a pQCT analysis of a cross-section of the forearm at measurement height. Color scale below the cross section indicates the range of the calculated bone density

Inclusion criteria were defined as female postmenopausal patients at least 50 years of age who sustained a single distal radius fracture (AO-type A3 or C1–3; see Fig. 1a) requiring extrafocal k-wiring and mounting of an external fixateur. Exclusion criteria were malignoma or medical treatment with bisphosphonates or other drugs affecting bone turnover.

The participants were assigned to placebo or verum group following the randomization sequence created by the study biostatistician. All study personnel remained blinded to the subject treatment assignment. Randomized subjects were assigned to receive either estrogen or a corresponding placebo plaster. Estrogen was applied via a transdermal plaster containing 4 mg estradiol (Estramon® Uno 50**,** Hexal AG**,** 83607 Holzkirchen, Germany). Four plasters were handed over to the patient. Each plaster was applied for 1 week (this corresponds to a daily dose of 0,571 mg) resulting in a total treatment time of 4 weeks. The proper application was demonstrated to the patient when the first plaster was set. The patients of the control group received the corresponding placebo plaster which could not be distinguished from the estradiol plasters.

Before surgical treatment of the fracture, and 4 weeks later, blood samples were collected and estrogen levels were measured. After taking the first blood sample, the patients both groups received daily 1000 mg calcium and 880 IU vitamin D (25-[OH]-vitamin D) over a period of 6 weeks to avoid any influence of a lack of calcium or vitamin D. The calcium, parathyroid hormone (PTH), vitamin D and alkaline phosphatase serum levels were analyzed before surgical treatment and 6 weeks postoperatively. The calcium, parathyroid hormone (PTH) and vitamin D values in the serum of patients of both groups have already been published in a study by Kolb et al. (2013) [16].

The present study was approved by the ethics committee of the University Medical Center Hamburg-Eppendorf (approval number: AZ 2252).

### Surgical intervention

Within an interval of 48 h after trauma, patients were surgically treated by k-wiring (two 1.6 mm titanium k-wires) and mounting of an external fixateur (HoffmannTM Fixateur, Stryker Howmedica, Mahwah, NJ, USA). According to our standard clinical care, X-ray controls in anterior–posterior and lateral projections were acquired prior surgery, postoperatively and after two and 6 weeks (see Fig. 1b and c). The X-ray controls only served to verify the correct implant position and not for tracking any kind of callus index. The external fixateur and the k-wires were removed 6 weeks after the surgery.

### pQCT measurements

pQCT allows the differentiation between cortical and trabecular bone. Because of the larger area, the trabecular bone reacts faster to changes in bone metabolism than the cortical bone. Bone loss or the success of a therapy can therefore be demonstrated at an earlier stage and with greater significance with pQCT than with methods that cannot distinguish between cortex and cancellous bone [15]. We applied therefore the pQCT technology to score the success of the post-surgical treatment with transdermally applied estrogen.

pQCT scans, (XCT-2000; Stratec Medizintechnik, Pforzheim, Germany) of the fractured and contralateral non-fractured distal radius were performed within defined intervals: postoperatively, after two and 4 weeks as well as after removal of the external fixateur and k-wires after 6 weeks. The device was calibrated using a standard phantom and a cone phantom provided by the manufacturer. The settings selected for the pQCT analysis were adopted according to the manufacturer’s recommendations as previously described [16–20].

The forearm length was defined as the distance between the ulna styloid process and olecranon. Measurements were taken while forearm was supinated and elbow flexed at 90 degrees. A 2-mm-thick single tomographic slice with pixel size 0.59 × 0.59 mm was taken directly in the fracture plane of the distal radius using the standard mode (see Fig. 1d). Image processing and calculation were performed using the manufacturers software package CXCT550 (version 5.50D). The bone mineral density (BMD) was defined as mean density of the total cross section.

The k-wires which were used to position and fix the bone fracture after surgery interfered with the pQCT measurements. Control measurements at the − 4% position from the fractured site could also not been taken into account, since the fractures of the patients were at variable positions of the radius, and thus the − 4% sites were at different positions dependent on the fractured site. Thus, the k-wire fixatives precluded a reliable quantitative analysis during the initial and early bone healing process. pQCT measurements at the fractured site and at the − 4% control position were therefore first examined 6 weeks post-surgery and after the k-wires were removed and analyzed together with the corresponding X-ray images.

### Determination of calculated bone density (CALCBD)

Based on the protocol of the manufacturer, the calculated bone density (CALCBD), which determines the total and cancellous bone density was acquired in three steps:

1. A region of interest (ROI) was defined, which was the region tight to the outer contour of the radius and was individually determined by us on the device.
2. Within this ROI, based on a soft tissue threshold (280 mg/cm^3^), each pixel outside the bone window was defined and removed. This left the bone part of the ROI from which the total area and the total density (CALCBD) was derived.
3. The total area was concentrically divided in the outer 55%, which was defined as the cortical and subcortical area, and the inner 45%, defined as trabecular area.

The total CALCBD levels were determined at different times and at different sites of the arm. These sites were (i) the fractured site which was determined on the basis of the X-ray images, (ii) a position which is 4% of the bone length of the radius above the fracture gap (− 4% level) and (iii) the corresponding position of the non-fractured healthy arm. Measurements were taken from estrogen treated and placebo treated control patients. The − 4% position of the fractured arm were only taken into account 6 weeks after the surgery and after both k-wires and fixative, since they interfere with measurements. The measurements on the healthy side were performed directly postoperatively (zero weeks) and after 6 weeks.

### Determination of cortical bone density (CORTBD)

The cortical bone density (CORTBD) is a measure of cortical bone structure in the diaphysis. For its determination, all pixels within the ROI with a lower density than the threshold (here 710 mg/cm^3^) were removed. From the remaining pixels, the cortical area and cortical mineral density were computed.

### Examination of the serum levels of diagnostic molecules

**Fig. 2:**
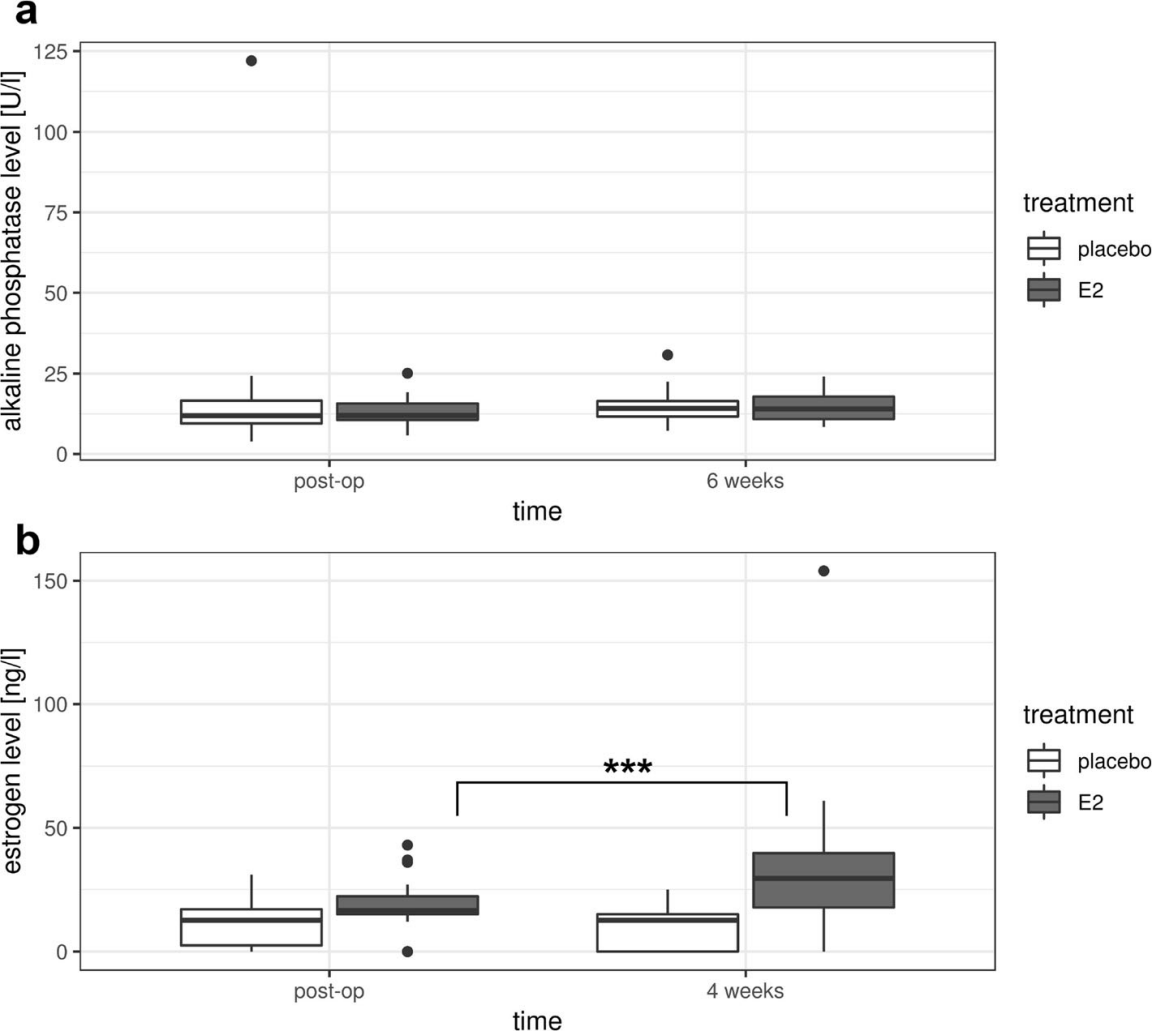
**a:** Bone-specific alkaline phosphatase activity levels of estrogen-treated patients (E2) and patients of the placebo control group (placebo). Bone-specific alkaline phosphatase activity levels were measured in blood samples of the patients directly after the surgery (post-op) and 6 weeks (6 weeks) later. Error bars show standard deviation. **b:** Estrogen levels of estrogen-treated patients (E2) and patients of the placebo control group (placebo). Estrogen levels were measured in blood samples of the patients directly after the surgery (post-op) and 4 weeks (4 weeks) later. Note the significant increase (p^***^ = 0.0003) of estrogen levels in response to transdermal estrogen treatment over a period of 4 weeks. n (E2) = 31; n (placebo) = 45

Bone-specific alkaline phosphatase activity (U/l) in the blood samples of patients was measured immediately after the surgery and 6 weeks later (see Fig. 2a), at the Central Laboratory of the University Medical Center Hamburg-Eppendorf as described before [21]. Calcium (mmol/l), parathyroid hormone (PTH) (ng/ml) and vitamin D (μg/l) serum levels were analyzed before surgical treatment and 6 weeks postoperatively [21].

### Statistics

Statistical analysis was performed using a student’s t-test for age comparison and for the data comparing estrogen-treated and placebo patients. Dependent on the outcome variable, either linear regression or mixed-effects models were used to analyze the effect of the different independent variables. The significance level was set to alpha = 5% for all statistical tests. All analyses were performed with the statistics software R (version 3.4.0, www.r-project.org) using the R-package lme4 for the mixed-effects models. The power analysis performed for the patient groups E2 (*n* = 31) and placebo (*n* = 45) with alpha = 5% and power of 80% showed that a medium effect size could be detected (d = 0.59) (G*Power version 3.1.9.4).

## Results

A total of 76 patients with complete clinical datasets were included in this double-blinded randomized study. Of those, 31 patients (mean age: 71.13 years ±11.99 years) were treated with estrogen and 45 patients (mean age: 75.62 years ±10.47 years) were left in the placebo group. The age difference between patients of the two groups was not statistically significant (*p* = 0.1006; see Table 1). The dominant arm was fractured in 29.03% of the patients of the estrogen treated group, and in 53.33% of the patients of the placebo group (see Table 1).

**Table 1:** Baseline characteristics of the populations

***Parameters***	***Placebo (n = 45)***	***E2 (n = 31)***
Age range [years]	55–94	51–89
Age mean [years] ± SD	75.62 ± 10.47; *p = 0.1006*	71.13 ± 11.99; *p = 0.1006*
Fractured dominant arm [%]	53.33	29.03

*E2* estrogen treated patients; *n* number of patients; *SD* standard deviation

To determine the effect of transdermal estrogen treatment, we first examined the estrogen levels in blood samples of patients immediately after the surgery and 4 weeks post-surgery, respectively. Figure 2b shows box plots of the estrogen levels in the blood of estrogen-treated and placebo-treated patients immediately after surgery and 4 weeks later. The estrogen values of all patients prior to the estrogen application or the placebo-treatment were in the range of ≤ 30 ng/l, indicating that all individuals were in post menopause [21]. Four weeks after the surgery, the estrogen concentration was significantly increased only in the estrogen-treated patients, while no significant increase was observed in the patients of the placebo group (Fig. 2b). The results indicate that the 4 weeks of transdermal estrogen-treatment increased the estrogen levels significantly (increase between 12.0 and 25.0 ng/l) in 18 patients and only slightly in the others. In contrast, the estrogen values of the untreated control patients remained low, i.e. < 30 ng/l. In the estrogen-treated group, 28 out of 31 patients had estrogen blood levels in a range commonly observed in premenopausal women. Two patients had significantly higher estrogen levels than normal and in one case, the level was lower because the patch had been fallen off during the process of the study (see Fig. 2b).

We compared the healing process by measuring the total calculated bone density (CALCBD) and cortical bone density (CORTBD) by peripheral quantitative computed tomography (pQCT) at three different sites of the patients as described in the Methods section.

### Calculated bone density and cortical bone density in estrogen versus placebo treated patients

**Fig. 3:**
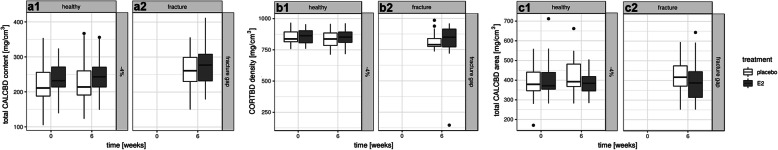
**a1** and **a2:** CALCBD levels at different time points and at different heights of the radius. **a1:** Measurements at − 4% of the total length of the radius on the healthy side. Measurements were taken after zero and 6 weeks. The isolated points represent two outliers. **a2:** Shows the measurements at 6 weeks after removal of the external fixateur and the k-wires on the fractured side (n (E2) = 31; n (placebo) = 45). **b1** and **b2:** CORTBD density levels at different time points and at different heights of the radius. **b1:** Measurements at − 4% of the total length of the radius on the healthy side. Measurements were taken after zero and 6 weeks. **b2:** Shows the measurements at 6 weeks after removal of the external fixateur and the k-wires on the fractured side (n (E2) = 31; n (placebo) = 45). The isolated points represent three outliers. **c1** and **c2:** CALCBD area levels at different time points and at different heights of the radius. **c1:** Measurements at − 4% of the total length of the radius on the healthy side. Measurements were taken after zero and 6 weeks. The isolated points represent three outliers. **c2:** Shows the measurements at 6 weeks after removal of the external fixateur and the k-wires on the fractured side (n (E2) = 31; n (placebo) = 45). The isolated point represents an outlier. Median values are shown

The k-wires used to fix the bone fracture after surgery interfered with the pQCT measurements and thus, they precluded a reliable quantitative analysis during the initial and early bone healing process. For the fractured side, pQCT measurements were therefore only performed 6 weeks post-surgery when both the external fixateur and the k-wires were removed. At that time point, we measured the total calculated bone density (CALCBD) of the estrogen and placebo treated patients. Figure 3a1 shows total CALCBD content of the healthy arm at different time points of the estrogen-treated patients and the patients of the placebo group (Fig. 3a1). Immediately after the surgery, the median CALCBD value of patients of the placebo group was 227.40 mg/cm^3^ and remained in a similar range after 6 weeks of fracture healing. The corresponding values of the estrogen-treated patients were not statistically different (*p* = 0.4388 and *p* = 0.5688, respectively) immediately after surgery and after the 6 weeks healing period.

Figure 3a2 shows the CALCBD content at the fractured site of the patients 6 weeks after the surgery. It was 253.90 mg/cm^3^ at the fraction gap of the placebo group and increased to 277.74 mg/cm^3^ in the estrogen-treated patients (*p* = 0.5565). Figure 3b1 shows the CORTBD density also from the healthy arm at different time points, postoperatively and after 6 weeks of surgery at the − 4% position. The median CORTBD density of the placebo treated patients at the − 4% position decreased from 834.96 mg/cm^3^ immediately after surgery to 787.92 mg/cm^3^ (*p* = 0.9190) after 6 weeks. The corresponding values were 849.42 mg/cm^3^ immediately after surgery and 794.23 mg/cm^3^ 6 weeks post-surgery (*p* = 0.3154). Six weeks after the surgery, the median CORTBD values at the fractured site of the placebo treated patients was 752.32 mg/cm^3^ and 852.29 mg/cm^3^ (*p* = 0.9487) for the estrogen-treated patients (Fig. 3b2). Thus, both the CALCBD and the CORTBD were not significantly different in estrogen-treated patients and the placebo group.

Figure 3c1 summarizes the CALCBD measurements at − 4% control position from the healthy arm of the patients at different time points, postoperatively and 6 weeks after the surgery. The median total CALCBD area on the healthy side of the placebo group immediately after the surgery was 389.88 mg/cm^3^ as compared to 399.33 mg/cm^3^ after 6 weeks (*p* = 0.2852). The corresponding values of the estrogen-treated patients were 375.77 mg/cm^3^ and 395.90 mg/cm^3^ (*p* = 0.3475), respectively. Figure 3c2 shows the total CALCBD area from the fractured side after 6 weeks of surgery at the fracture gap. The median value of the patients of the placebo group was 419.36 mg/cm^3^ as compared to the 394.70 mg/cm^3^ of the estrogen-treated patients (*p* = 0.8036). Again, there is no statistically significant difference with respect to callus BMD as measured by pQCT between the two patient groups. Also, no significant effect on bone density parameters in response to the transdermally applied estrogen could be detected in the unfractured arm. Nevertheless, the data suggest, since the *p*-values are still rather small (see above) there is a notable tendency towards better callus BMD of the patients who received transdermally applied estrogen.

**Fig. 4:**
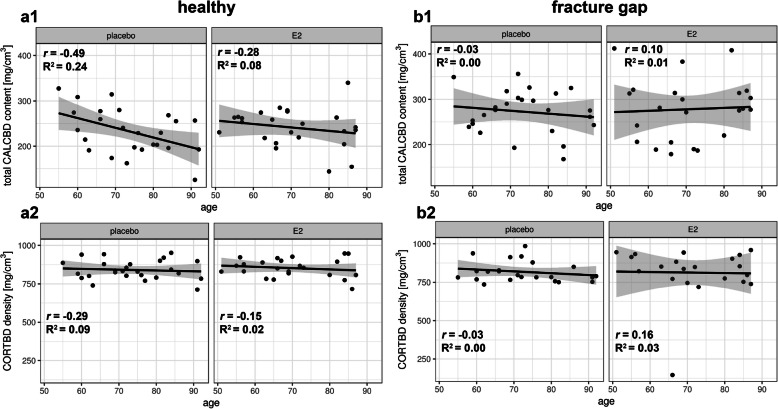
Shows scatterplots representing measurements of the CALCBD content (**a1** and **b1**) as well as the cortical bone density (CORTBD) (**a2** and **b2**) of the healthy arm (**a1** and **a2**) compared to the fractured arm at the height of the fracture gap of the radius (see **b1** and **b2**); the scatterplots represent the measurements with (right side) and without treatment (left side) of estrogen. **a1:** shows total CALCBD levels of the healthy side of individual patients of different ages with bone fractures on the opposite side. **a2:** shows the CORTBD density measurements during bone healing. **b1:** shows total CALCBD levels of the fracture gap of individual patients of different ages. **b2:** shows CORTBD density in the fracture gap. The gray shading shows the 95% confidence interval. n (E2) = 31; n (placebo) = 45. R^2^ represents the coefficient of determination; *r* represents the correlation coefficient

We next asked whether there is an age-dependent effect of estrogen on callus BMD as measured by pQCT because of the reduced sensitivity to estrogen with age [22]. Figure 4 summarizes the data from CALCBD (Fig. 4a1 and b1) and the CORTBD analyses (Fig. 4a2 and b2) of the healthy (Fig. 4a1 and a2) and fractured (Fig. 4b1 and b2) arm of postmenopausal women of different ages. Figure 4a1 shows the total CALCBD levels of the healthy arm of individual patients of different ages. Mixed effects model calculations indicated that callus formation tends to decline in both estrogen treated and untreated postmenopausal patients during ageing. Although the scatter range was large, this effect was statistically significant (p^**^ = 0.0014) but independent of the estrogen treatment (Fig. 4a2). Figure 4b1 shows the total CALCBD level at the fracture gap of individual patients of different ages, indicating that callus formation at the fractured site follows a declining trend in the placebo group, whereas a trend in the estrogen treated patients towards increased callus formation was again notable. Furthermore, CORTBD density measurements at the fracture gap showed a tendency to decrease with the age of the placebo group patients, whereas a stable and consistent status prevails in the estrogen treated patients (Fig. 4b2) as reflected by the rather small *p*-values.

### Alkaline phosphatase serum levels are not affected by estrogen treatment of the patients

Bone-specific alkaline phosphatase (ALP) activity is a diagnostic maker for neotissue mineralization during bone growth and fracture healing [23]. Thus, in addition to the morphological studies on bone healing, we also measured ALP activity in the blood serum of the patients. These measurements were performed on patients with and without estrogen treatment both immediately after surgery and 6 weeks later. In agreement with morphological studies reported above, there was no significant effect of the estrogen treatment. Untreated patients of the placebo group had an average ALP value of 51.37 U/l (standard deviation: 53.49 U/l) and 49.53 U/l (standard deviation: 52.47 U/l) after surgery and 6 weeks later, respectively. The corresponding ALP values of the estrogen treated patients were 28.15 U/l (standard deviation: 33.88 U/l) and 30.12 U/l (standard deviation: 32.19 U/l). The similar ALP values suggest that neotissue mineralization during fracture healing is not affected in response to the transdermally applied estrogen. Taken together, both the morphological measurements and the enzymatic ALP activity showed no significant effect of estrogen treatment on bone fracture healing.

## Discussion

Even though transdermal estrogen treatment increased the systemic estrogen levels of the patients, we did not observe an effect of this treatment on an early phase of bone healing, namely callus BMD as measured by pQCT, in postmenopausal women. Several lines of preclinical evidence have suggested that estrogen plays an essential physiological role during bone development and the bone remodeling process [4]. In rats, the administration of estrogen in a sustained way was shown to be able to reverse the decline in bone strength and to re-establish the bone quality [8, 24, 25]. Furthermore, long-term ovariectomized rats showed impaired callus BMD [26]. Moreover, histomorphological analyses demonstrated a delay in fracture callus healing with poor development of mature bone in these animals [27]. These findings suggested a possible role of the hormone in callus formation as the remodeling of the fracture callus is essential during this process [4, 28]. It has also been demonstrated that cells involved in bone healing such as osteoblasts, osteocytes and osteoclasts express functional estrogen receptors, ER-alpha and ER-beta, and that ER alpha supports callus formation and thus bone healing [29]. These receptors are also expressed in bone marrow stromal cells, the precursors of osteoblasts. In addition, Boden et al. (1989) [30] reported that rat estrogen receptor genes are expressed in fracture callus, implying that estrogen could play a role in the normal fracture healing process. Finally, it was shown in a mouse model that estrogen plays a crucial role in fracture healing [4], and that estrogen may enhance the fracture healing process in animal models such as rabbits [9]. Collectively, these findings suggested that estrogen treatment could be indeed beneficial for postmenopausal patients with osteoporotic fractures [31].

However, there are reports on severe side effects of estrogen treatment of human. High doses of estrogen can cause incident coronary heart disease, stroke, venous thromboembolism, breast cancer, colorectal cancer, endometrial cancer or hip fractures [32–34]. It has recently been shown that estrogen-alone hormone therapy does not increase the risk of breast cancer in postmenopausal women as outlined in an updated report of the Women’s Health Initiative (WHI) estrogen-alone trial [35]. Doses of 0.625 mg/day of conjugated equine estrogen (CEE) were applied to 10,739 post hysterectomy women showing that they caused other adverse reactions [36]. This dose was about twice as high as applied in the animal model organisms where beneficial effects on bone fracture healing were reported, but as observed in humans, strong side effects were also observed [4].

Studies analyzing the dose responses of estrogen with respect to bone healing, its dose-dependent side effects and clinical studies with lower doses of estrogen on post hysterectomy women have not yet been reported. To avoid any potential estrogen-dependent complications [32–34] in our study, we used a sufficiently low estrogen dose to reach the endogenous systemic level of estrogen equivalent to the level in premenopausal women. This was achieved by applying a weekly transdermal dose of 4 mg estradiol over a period of 4 weeks. This treatment increased the endogenous estrogen level in the blood of the patients from about 16 ng/l to a blood concentration of ≤30 ng/l estrogen, corresponding to the endogenous estrogen level of women during the first half of the menopause cycle [37].

Our findings showing that there is no statistically significant beneficial effect of estrogen treatment on callus formation, as established by BMD measured by pQCT, are in apparent contrast to observations with preclinical models. They show that estrogen administration, although at much higher doses however, strengthens indeed the properties of bones of ovariectomized animals after femoral fracture [4, 8–13, 38]. However, there are no studies yet which determine the minimal dose of estrogen that causes a beneficial effect on bone healing in the preclinical model organisms without producing the known side effects of estrogen. It could well be that a high estrogen dose as applied to the preclinical models would also have beneficial effects on human bone fractures, but causes otherwise adverse side reactions. It is noteworthy that mechanostimulation by low-magnitude high frequency vibration was shown to provoke anabolic effects on the intact skeleton of both mice and human. This effect is estrogen-dependent and shown to be mediated primarily via the estrogen receptor alpha in mice, and vibration-induced effects on fracture healing in combination with estrogen treatment have also been shown to improve the osteopenic bone structure and increased the bone stiffness in preclinical studies [13, 39–41]. Thus, it is possible that such a combination of treatments rather than estrogen treatment alone could improve the healing of bone fractures by positively affecting callus BMD as measured by pQCT.

We observed that callus formation after bone fracture was age-dependent. The CALCBD levels decreased with the age of the patients, i.e. the on average 60-year-old patients had a CALCBD level of 250 mg/cm^3^, whereas the CALCBD level of 90-year-old patients was about 20% lower. This finding suggests a tendency for the reduction of callus formation with increasing age.

Our study shows that increasing the levels of estrogen in postmenopausal women to premenopausal levels have no significant effect on callus BMD as measured by pQCT. Possible reasons for this observation could be insufficient acuity of the chosen measurement protocol, and/or an insufficient dose of estrogen applied. Alternatively or in addition, there could be an age-dependent reduction of estrogen receptors in the hormone-receiving bone cells, representing a limiting factor for bone regeneration in postmenopausal women. One additional reason could be that estrogen was transdermally administered and that a more local administration may support callus BMD formation as a prerequisite for better bone fracture healing.

We note that our clinical study concerning the effect of estrogen treatment after bone fractures has some limitations. First, since we examined the increase of estrogen levels only 4 weeks after the surgery, we do not know when the increase of the estrogen level occurred. It is therefore possible that the effective estrogen level was not reached when necessary for enhancing the BMD. Furthermore, the estrogen levels applied to mice, where a beneficial on callus formation was observed [4] was about three times as high as applied to human patients. Such as estrogen concentration would already have severe side effects [32–34] in human and thus, it could not be applied to patients in our study. Thus, it is possible that positive effects on callus BMD and subsequently on bone healing cannot be achieved without the risk of severe side effects [32–34] which were not examined with the preclinical animal models [4, 8–13]. Secondly, additional methodology such as X-rays for bony bridging, MRI for callus development as well as other functional analysis would need to be applied to definitely rule out that estrogen has no beneficial effect on callus BMD, as established here by pQCT, and subsequently on bone fracture healing in human patients.

## Conclusion

Raising the level of estrogen in postmenopausal women to the systemic level in premenopausal women has no significant beneficial effect on callus BMD as measured by pQCT.

## Supplementary information

**Additional file 1.** CONSORT guidelines. The study was performed according to the CONSORT guidelines. (DOC 220 kb)

## Data Availability

“The datasets supporting the conclusions of this article are included within the article (and its additional files). Additional datasets used for the current study are available from the corresponding author upon request.”

## Abbreviations

ALP: Alkaline phosphatase
BMD: Bone mineral density
CALCB: Calculate bone density
CORTBD: Cortical bone density
E2: Estrogen
ER: Estrogen receptors
OBs: Osteoblasts
OCs: Osteoclasts
PTH: Parathyroid hormone
pQCT: Peripheral quantitative computed tomography
ROI: Region of interest
